# Supplementary material for: Implicit GPS-based bicycle route choice model using clustering methods and a LSTM network
Source: PLoS One. 2022 Mar 17;17(3):e0264196. doi: 10.1371/journal.pone.0264196 (PMC8929588; doi:10.1371/journal.pone.0264196)
Supplement: S1 Appendix — (PDF) [file pone.0264196.s001.pdf]

A MonResoVelo

The *MonResoVelo* data-set contains 5000 GPS tracks but only 2800 tracks remains after removing the tracks having a too long detour and the noise found by DBSCAN. We shows the results of our intermediate model using an oracle on Fig 13 and the results of our final model in Fig 14.

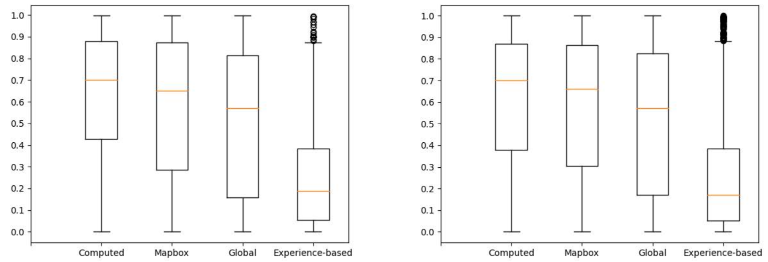

Fig 13. Box-plots of the distance between the experienced tracks and the four other types of tracks. The experience-based ones are generated using an oracle. The left box-plot is for the 20% experience based tracks removed from the clusters, the right one for the 80% remaining.

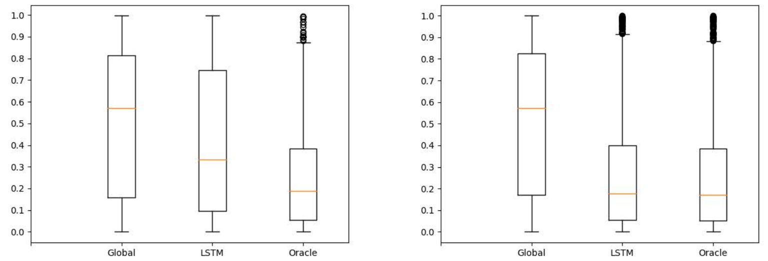

Fig 14. Box-plots of the distance between the experienced tracks and the experience-based tracks. The left one shows the testing data-set and the right one the training data-set.
