# Supplementary material for: Implicit GPS-based bicycle route choice model using clustering methods and a LSTM network
Source: PLoS One. 2022 Mar 17;17(3):e0264196. doi: 10.1371/journal.pone.0264196 (PMC8929588; doi:10.1371/journal.pone.0264196)
Supplement: Attachment — Submitted filename: Response to reviewers.pdf [file pone.0264196.s002.pdf]

Response to reviewers

Lucas Magnana, Hervé Rivano and Nicolas Chiabaut

November 2021

We want to start by thanking the two reviewers for their comments. We have answered each one of them with the greatest attention, and have modified the paper in several places. The introduction has been completely reworked to make our objectives and the positioning of our work in the current literature more explicit. We begin by responding to the comments raised by the two reviewers, then respond to the comments of reviewer 1 and finally to those of reviewer 2. The edits are written in red in the revised manuscript as well as in this letter.

1 General remarks

1. *R1: The authors should list the specific contribution of this work.*
   R2: What are the practical contributions of this study?

   AR: Both reviewers pointed out that the contributions of our work remained unclear in the previous version. We try to precise the insights provided by our paper. First, we clustered the GPS tracks in order to group tracks having several road segments in common. It allows an enrichment of the data as it identify the road segments that cyclists use the most. This enrichment is used to generate a cycling path between a pair of O/D. Our method consists in modifying the road graph of the city using a cluster, and to execute a shortest-path algorithm on it to create cycling paths. The paths created therefore use the data only, and no external factors unlike classical route choice models. We trained a LSTM to select the cluster to use to create the most accurate cycling path from a pair of O/D. Finally, we compared the similarity between the initial GPS tracks and tracks generated with different methods (including ours). This comparison allows to measure how well our method allows to generate tracks simulating the behavior of cyclists. We listed these contributions at the end of the introduction in the revised manuscript.

   Edit in Section 2:

   The main contributions of our work can be summarized as follows:

   - We identify cyclists' preferred roads by clustering GPS tracks passing through the same road segments.
   - We develop a method to create a cycling path between a pair of O/D using a cluster of GPS tracks.
   - We train a LSTM neural network to choose the cluster to use to create the most accurate cycling path from a pair of O/D.
   - We compare the similarity between GPS tracks generated by cyclists and tracks created using several methods.

   Fig 1 shows the structure of our method and model.

2. *R1: Page 1, Introduction: The distribution of this part is unreasonable, and the introduction of LSTM is relatively less. The content of literature review is few. Some references provided are outdated. And the research question and gap are not clear.*
   R2: Some recent studies should reviewed, for example, see: On the development of a semi-nonparametric generalized multinomial logit model for travel-related choices. PloS one, 12(10), e0186689. Radial Basis Function Neural Network with Localized Stochastic-Sensitive Autoencoder for Home-Based Activity Recognition. Sensors, 20(5), 1479.

   AR: In the previous version, the introduction was unreasonably distributed, and we introduced the LSTM in the Section 6. To solve both of these issues, we moved the presentation of the LSTM networks from Section 6 to Section 2. This allows the reader to understand from the introduction which methods are used in our study.
   We have also reworked the structure of Section 2 and added a paragraph in order to make the research question and gap clearer. Several types of cycling data exist. The more accessible are data from bike-sharing systems, but this type of data only contains the position and time of the points of origin and destination. Statistical, machine learning and deep learning algorithms are usually used on this kind of data to predict the BSS state at a given time at different scale (the whole system, groups of stations or stations). But these studies only focus on O/D pairs without taking into consideration the paths taken by the users, as the data provided by the BSS are not precise enough to do so. Studies analyzing and modeling paths used by cyclists have to use more detailed data. Historically using surveys, the democratization of GPS sensors has allowed the constitution of GPS tracks data-sets which are mainly used by this type of studies today. The classical way to analyze and model the cyclists' behaviors is to create a discrete route choice model using individual observations. It is done by generating a choice set of paths for each observation, and choosing external factors to describe the generated alternatives. A logit is then trained to choose the best option between the alternatives in the choice sets. The main issues of the discrete route choice models are that the choice sets of paths generation along with the factors used are sources of bias. Our objective is to create a bike route choice model without using external factors or choice sets of paths. We replace them by learning algorithms to create a bike route choice model that combines the predictive performance of the algorithms used on BSS data with the completeness of GPS data.
   Finally, we have refreshed the bibliography. Older publications have been replaced by more recent ones, and we have added recent papers (including those proposed by reviewer 2) in particular in the presentation of LSTMs to justify our remarks.

   Edit in Section 2:

   Neural networks have proven their effectiveness in prediction in recent years. Recurrent Neural Networks are NNs capable of handling time-dependent and sequential data. They have been widely used in fields using temporal data such as speech recognition, language modeling, translation or image captioning. LSTM networks are RNNs specially designed to handle long sequences. Used on BSS data, LSTM networks outperform flows prediction's performances of classic statistical, deep and machine learning methods on station-level. In 2019, Pan et al used a LSTM network on data from Citi Bike System Data to predict the number of bikes rented and returned in the BSS system. They divided each day in 24 time steps and their model is capable of predicting the number of bikes rented and returned at each station at each time-step. The LSTM network outperforms the predictive capabilities of a classical neural network in that case, as the classical neural network does not take into account the sequential aspect of the data. However, studies studying flows in BSS systems only predict the behavior of BSS users, which may not be representative of the total number of cyclists. Moreover, the origin and destination points of BSS users are limited by the stations. It is probably a coarse and biased sampling of the space.
   LSTM networks can also be used on dockless BSS systems that are gradually being implemented in the world. These systems offer the advantage of not restricting the origins and destinations

of their users as bicycles can be picked up and dropped off anywhere in the city. Recent studies are dividing the city in several parts and use LSTM networks to predict the bike distribution or the trip production and attraction in each one of the part.

Edit in Section 2:

This paper aims to develop an implicit bike route choice model which use learning methods instead of external factors and do not require the generation of choice sets of paths. The objective of our model is to generate a cycling path when given an origin/destination pair. The model learns from previous individual observations to create paths that simulate cyclists' behaviors. The learning creates implicit representations of the data which replace the external factors used in the more classical methods. Our model uses learning tools classically used on BSS data on GPS data. This allows to keep the completeness of the GPS tracks and to make predictions of cycle tracks using only observed data, thus limiting the biases created by the generation of the choice sets of paths and the choice of external factors.

2 Reviewer 1

1. *I suggest that the limitation of this work should be discussed.*

   AR: The conclusion of the previous version mentioned possible future researches but not about the limitations of our work. We identified three major ones. The first comes with the data we used. The GPS tracks have been generated by only forty cyclists doing commuting trips. This sample is not representative of all cyclists, and the redundancy in the routes facilitated the clustering and thus the training of the LSTM. The second one is intrinsic to our method. Since external factors are replaced by learning algorithms, our method can simulate cyclists' behavior but not explain it. Finally we did not use the temporal properties of the GPS tracks in order to facilitate the setting of the learning algorithms. In the revised manuscript, we created a section named "Discussion" on which both of the limitations and the possibility in future researches are discussed.

   Edit in Section 8:

   A first limitation of our work is the dataset we used. We were unable to find a massive public dataset of GPS tracks generated by cyclists. Therefore, we used a private one that we already owned. It gathers GPS tracks of a small group of cyclists who mainly made commuting trips. It is thus made up of tracks that are similar to each other, facilitating the clustering work. *In future research [...] with a low silhouette score.*
   Secondly, our method does not explain the causes of the cyclists' choices and preferences it identifies. These identifications depend on the representations learned by the learning algorithms which are by nature implicit.
   Finally, we did not use the spatial properties of the GPS tracks, as we wanted to focus only on the spatial ones. In future works, using temporal properties could improve the performances of the learning algorithms and therefore the accuracy of the generated cycling paths.

2. *Page 1, Abstract: It is unclear to show the overall idea and purpose of this paper.*

   AR: We regret that the reviewer consider that the overall idea of the paper was not clear. We created a route choice model which differs from classical ones by not using choice sets of paths and replacing external factors with learning algorithms. The goal is to create tracks that simulate the cyclists' behavior using only GPS tracks previously generated by them. We modified the abstract to make it clear.

   Edit in Section 1:

These policies having a cost,understanding and predicting the behavior of cyclists has become a necessity in order to optimize them. Classical methods analyzing cyclists' route choices use external factors and generated choice sets of paths along with a logit model to create a discrete route choice model. Nevertheless, few studies focus on the predictive capacity that this type of model can offer. In this paper, we developed a prediction-centered bicycle route choice model. Our model is created without using external factors or choice sets of paths as in the more classical methods. The idea of our method is to use deep and machine learning algorithms on GPS tracks. These algorithms learn representations from the data which replace explicit factors.

3. *Page 4, in section 3.1: Two data-sets are used. However, there is no specific description of the two types of data collection events and processes. It is necessary to add the example of these two data-sets and give the standard of screening data.*

   AR: The two data-sets we used were not formally presented. We added the presentation of both of them as well as the modifications we have made to them in the revised manuscript. Each presentation includes the collection method, the period of collection, the number of tracks and the data format.

Edit in Section 3.1:

Despite the increasing number of GPS sensors and the number of studies using these kinds of data, only a few data-sets of biking GPS tracks are publicly available, and those are of small or medium size. Two data-sets are used in our study:

- A private set of GPS tracks coming from a multidisciplinary research project in University of Lyon called *Veleval*. Forty volunteer cyclists were asked to do commuting trips by bike from May 2016 to October 2019 in Lyon and Saint-Etienne, France. Each cyclist carried a GPS tracker during his trips. The result of this project is 2535 GPS tracks contained in GPX files. Each GPS track t is a sequence of N ordered points $t = \{p_1, ..., p_N\}$ with each point $p_t = (lat_t, lon_t, time_t)$ being made up of a latitude value, a longitude value and a timestamp.
- An open data-set provided by Montreal, Canada, collected by their *MonResoVelo* application (public). MonResoVelo is a smartphone application that cyclists could use while biking to be tracked during their trips. Data collected by the city were then used to improve the development of cycling facilities. 4881 GPS tracks from this application are public. They have been generated by cyclists from June 2013 to October 2015 and are contained in a JSON file. Each track t is a sequence of N ordered points $t = \{p_1, ..., p_N\}$ with each point $p_t = (lat_t, lon_t)$ being made up of a latitude value and a longitude value. Other information such as the departure and arrival time are available but we only used the GPS points. The tracks are already mapmatched and anonymized.

We decided to ignore the temporal properties of the tracks and to only focus on the spatial ones, because it makes the setting of the learning algorithms used later easier. We thus removed the timestamps from the *Veleval* project tracks. We used the Ramer-Douglas-Peucker algorithm on both datasets to decrease the number of GPS points per tracks. Fewer GPS points means shorter processing time. For the sake of brevity, only the Veleval data are presented in the paper. We preferred the Veleval dataset to the MonResoVelo one because it contains almost exclusively commuting trips. As our model has to create paths using an O/D pair, using a dataset of cyclists with a specific destination for each of their trips seemed more relevant. The purpose of tracks in MonResoVelo dataset can be among other things the practice of sports, or not even be defined. The results for MonResoVelo are nevertheless showed in Annex A. For the rest of this study, the cyclists' GPS tracks will be referred to as experienced tracks.

4. *We noted that 10% of the experienced tracks are shorter than the generated shortest path. However, no pictures or other source to support this conclusion.*

   AR: The 10% of experienced tracks shorter than the generated shortest paths were not justified in the previous version. We did not want to insist on this point because it is a simple side effect due to the imperfection of the road graph. We added a figure (Fig 2 in the revised manuscript) of the distribution of the detours to support our point about these tracks.

   Edit in Section 3.2: Fig 2 shows the distribution of distance differences between the experienced tracks and the generated shortest paths. The dashed red line shows the first experienced track longer than its shortest path. We can see that 10% of the experienced tracks are shorter than their shortest paths.

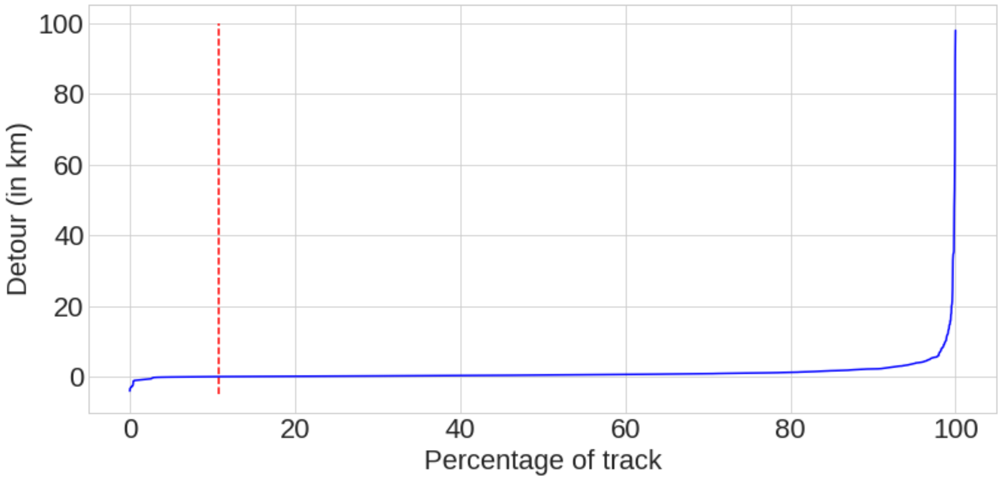

Figure 1: Distribution of distance differences between the experienced tracks and the generated shortest paths (sorted)

5. *Page 4, in section 3.3: First sentence, In order to verify that cyclists do not use the shortest paths. In the end of this paragraph, This is consistent with our hypothesis: cyclists would use the shortest route to reach preferred roads in the city center. Is there any contradiction?*

   AR: These two sentences were not in contradiction but were confusing. What we were trying to say is that cyclists do not use the shortest path to go to there destination but prefer to use more pleasant and/or safer roads. However, they tend to use the shortest path to access these preferred roads resulting in a mixture of preferred roads and shortest paths. We reworked these sentences in the revised manuscript to make it clearer.

   Edit in Section 3.3:

   To verify that the cyclists' tracks are different from the shortest paths between their origins and destinations, we generated two heat-maps. *The first one contains all the experienced tracks [...] preventing the visual distinction of the most used roads.* This is consistent with our hypothesis: cyclists are willing to use preferred roads, even if it means not using the shortest path between their origins and destinations. However, they seem to want to minimize the distance traveled to access these preferred roads. That's why we don't see preferred roads in the periphery. The

cyclists' routes would thus be a trade-off between specific road segments and shortest paths to access them.

6. *Page 5, in the part of quantitative comparison: Fig 3 shows the maximum detour reaches almost 100km. It is not shown in Fig 3 that the maximum detour reaches almost 100km. Also, it is not clear to see from the graph that the 10% of experienced tracks shorter than their computed tracks.*

   AR: The maximum detour of 100km is now shown in the figure showing the distribution of distances (Fig 2 in the paper). We removed the note about the 10% of experienced tracks shorter than their SP in the cumulative distribution function because this information is not relevant and can be confusing

   Edit in Section 3.3:

   The maximum detour reaches almost 100km (see Fig 2), but the CDF shows that 92% of detours are less than 2.5km.

7. *Page 8, in section 5.1: The cyclability coefficient takes values in [0,1], not]0,1].*

   AR: The interval of values was not right, we corrected it.

   Edit in Section 5.1: Thus, the cyclability coefficient takes values in $[0, 1]$, as the cell m has a coefficient of 1.

8. *Page 9, the Algorithm 1: The authors should give some explanation for each symbol and process of the algorithm.*

   AR: Some symbols and processes were not explained, we added explanations in the commentaries of the algorithm. All symbols are now explained in the revised manuscript.

   Edit in Section 5.3:

Algorithm 1: Road graph modification wrt a cluster

Data: A cluster c
Result: The road graph with modified weights
$\tau_c \leftarrow computeCells(c)$;
/* τ_c contains the set of cells through which routes in c pass */
for $e \in G$ **do**
  /* for each edge e in the road graph G */
  $\tau_e \leftarrow computeCells(e)$;
  /* τ_e contains the set of cells through which e pass */
  $\tau \leftarrow \tau_c \cap \tau_e$;
  $m \leftarrow mean(\mathcal{C}_c, c \in \tau)$;
  /* $\mathcal{C}_c$ is the cyclability coefficient of the cell c */
  $e.w \leftarrow e.w * (1 - m)$;
  /* e.w is the weight of e */
end

9. *The authors should to pay attention to English grammar, spelling, and sentence structure so that the goals and results of the study are clear to the reader. (Extra words appear in some sentences: Tracks created by our model are way more similar to the original GPS tracks)*

We have paid special attention to the grammar used in order to make our explanations and results as clear as possible. The revised manuscript should not contain sentences with extra words.

3 Reviewer 2

1. *There are already many machine learning methods, why use the DBSCAN and LSTM models?*

   Edit in Section 4.2

   AR: We feel sorry that the reviewer 2 did not find the choice of our models justified enough. We reworked the two sections dedicated to the explanation of our choices. We have paid particular attention to the explanation of the specificity of our data and the solutions provided by the chosen models.

   The relevance of using a clustering algorithm in a situation depends on the type of data (noisy or not, big or small data-set...) and the properties of the searched clusters (number, size, shape...). We want to create clusters to identify preferred road segments. To enable this identification, each track in the same cluster must share a certain amount of road segments with all other tracks. In Section 4.1, we developed a distance metric to quantify how much two tracks do or do not share road segments. Our clustering must therefore create groups of tracks that are dense with respect to our distance metric. This is the goal of density-based clustering algorithms, and in particular of DBSCAN, the most widely used of them.
   DBSCAN (Density-Based Spatial Clustering of Applications with Noise) is a density-based clustering algorithm that is able to find arbitrary shaped clusters and is well-suited to data with noise. Given a set of points and a distance, DBSCAN will group points closely packed together and mark as outliers points that have not a sufficient number of close neighbors. It does not require a predefined number of clusters, and has two parameters. We explain their meanings and how we set them up in Section 4.3. DBSCAN's properties make it a clustering algorithm adapted to our case. It generates groups of tracks that all share a certain amount of road segments which can then be used as preferred road segments. It also discards tracks that do not share enough road segments with others, allowing our model to use only tracks that represent minimally widespread behavior in our dataset.
   To confirm our intuition, we compared DBSCAN with k-means, the most known clustering algorithm. K-means is not density-based, and requires a number of clusters. It creates clusters of similar shapes and sizes, without taking noise into account. As we expected, some clusters created by k-means were large and contained tracks with no common road segments. In comparison, clusters created with DBSCAN all contain at least one preferred road segments visually noticeable.

   Edit in Section 6.1 A computed track is a set of points that follow each other in a particular order. We must therefore choose a classifier capable of handling sequential data. Recurrent Neural Networks (RNNs) are a type of classifier which have recently shown good results on this type of data. In addition to its input data, a RNN has a hidden state h_t which contains information about the data it previously processed. This allows it to take into account the temporal dependencies of the data it processes. We first tried to use a classical RNN for classifier. But it did not give us conclusive results, as some of our computed tracks were too long. Indeed, RNNs are confronted with the vanishing gradient problem which prevents them from learning long-term dependencies. The Long Short-Term Memory Neural Networks (LSTMs) have been developed to overcome this issue. LSTMs are a particular type of RNN which include a cell state in addition to the hidden state. The purpose of the cell state is to keep the information about the long term dependencies of the data. Fig 9 is a diagram showing a LSTM cell. LSTM

networks have shown better results than classical RNNs as computed tracks classifiers. We therefore chose this type of classifier for our final route choice model.

2. *Some dynamic factors for predicting the bike routes are not considered, for example, the weather condition, ect.*

   AR: Dynamic factors such as weather or social events are widely used when it comes to predicting travel in general, and this could very well have its place in a possible extension of our work. However, the goal of our project was to create a route choice model without adding external factors. We wanted to see if GPS tracks alone allows to model cyclists route choices. Moreover, we focused only on the spatial properties of the GPS tracks. In a future work, we could modify our method to add the temporal properties of the tracks and maybe add these kind of factors to improve the results. That said, we added this reflexion when talking about the limitation of our work in the "Discussion" section, and clarified the choice to not use the temporal properties when presenting the data we use.

   Edit in Section 3.1: We decided to ignore the temporal properties of the tracks and to only focus on the spatial ones, because it makes the setting of the learning algorithms used later easier.
   Edit in Section 8: Finally, we did not use the spatial properties of the GPS tracks, as we wanted to focus only on the spatial ones. In future works, using temporal properties could improve the performances of the learning algorithms and therefore the accuracy of the generated cycling paths.

3. *The proposed bike route choice model should be compared with the existing models in terms of the prediction accuracy.*

   AR:

   Comparing its results to those of existing models is a practice to be done as much as possible. To our knowledge, the only models that attempt to predict cyclists' behavior are discrete route choice models. We present these models in a paragraph in the introduction. This paragraph has been modified in the revised manuscript to make this presentation as clear as possible. Discrete route choice models need several observed paths used by cyclists. Choice sets of paths are generated to represent all the alternatives taken into account by the cyclists for each observed paths, and factors are chosen to describe these alternatives. A variant of the logit model is then used to assign a weight on each factor and calculate the probability of being chosen for each of the generated paths.

   Most of the studies developing a discrete route choice model are displaying the prediction accuracy of their calibrated modified logit. But the modified logit model aims to choose a path between a choice set. The prediction part of our model is the LSTM choosing a cluster from a shortest-path. The prediction part of a discrete route choice model is the logit choosing a path between several ones. Comparing these predictions would not be relevant. In any case most of these studies do not compare each other using the predictive ability of their models, but using the weights and t-stats of the different factors. As said in Section 1 of our paper, *studies developing discrete route choice models mainly focus on the factors influencing cyclists' choices and understanding their importance, but seldom analyze their model's route prediction capability.* To correctly answer your remark, we will talk about the few studies that do talk about their models' route prediction capability. There are two of them among the route choice models that we cite.

   The first study [2] develops a link-based bike route choice model. The predictions focus on two potential applications of a bike route choice model: predicting link-level bike volume and

measuring cyclist specific accessibility. The authors compare the methodological issues associated with previous models, then explain how their link-based model overcomes them. For link flows, they provide in addition numerical results which highlight the potential gains of time associated to their model. Our results focus on the similarity between the generated paths and the observations. We cannot compare them to predictions about the structure of the road graph.

The second study [1] use a similarity metric close to ours. The metric chosen by the authors uses the factors chosen for the logit which would make the comparison with our results complicated, but feasible in terms of scale. However their analysis only focuses on the similarity between the observations and the paths generated for the sets of choice. The study shows that there is at least one path close to the observation per set of choice, but their best model succeeds in finding the most similar path only 69% of the time. We lack the similarities between the observations and the paths chosen by the model to compare us.

Edit in Section 1:

To the best of our knowledge, the only existing models able to predict cyclists behaviors at a trip scale are the discrete route choice models. These models require the characteristics of several observed paths used by cyclists, collected in the past through preferences surveys, and more recently through GPS sensors. Choice sets of paths are generated to represent all the alternatives taken into account by the cyclists for each observed paths, and factors are chosen to describe these alternatives. A modified logit model is then used to assign a weight on each factor and calculate the probability of being chosen for each of the generated paths. A well-known issue associated with these models is that the set of all feasible paths is intractable and the actual choice sets of paths are unknown. As the model has to choose a route between the ones in a choice set, the accuracy of the model's behavior prediction is closely related to the quality of the sets. Moreover, choosing explicitly factors to describe the alternative paths allows only a partial analysis of route choices, as not all factors can be analyzed at the same time. This limitation is all the more present as the selected factors are practically the same in all studies. Designing new factors that can affect people's behavior is a difficult task involving multidisciplinary research. Today's factors are mainly infrastructural e.g. the number of turns or the proportion of bike facilities, global e.g. the length of the trip or the difference with the shortest path, dynamical e.g. weather or traffic volume, or social e.g. age, gender or car ownership. Finally, studies developing discrete route choice models mainly focus on the factors influencing cyclists' choices and understanding their importance, but seldom analyze their model's route prediction capability.

References

[1] M. Ghanayim and S. Bekhor. Modelling bicycle route choice using data from a GPS-assisted household survey. *European Journal of Transport and Infrastructure Research*, page Vol 18 No 2 (2018), Apr. 2018. doi: 10.18757/EJTIR.2018.18.2.3228. URL `https://journals.open.tudelft.nl/index.php/ejtir/article/view/3228`.

[2] M. Zimmermann, T. Mai, and E. Frejinger. Bike route choice modeling using GPS data without choice sets of paths. *Transportation Research Part C: Emerging Technologies*, 75:183–196, Feb. 2017. ISSN 0968090X. doi: 10.1016/j.trc.2016.12.009. URL `https://linkinghub.elsevier.com/retrieve/pii/S0968090X16302637`.
